# Technological and Functional Characteristics of Lactic Acid Bacteria from Traditional Serbian Cheeses

**DOI:** 10.3390/foods14010038

**Published:** 2024-12-26

**Authors:** Tijana Ledina, Jasna Đorđević, Milica Glišić, Nikola Čobanović, Marija Kovandžić, Snežana Bulajić

**Affiliations:** Department of Food Hygiene and Technology, Faculty of Veterinary Medicine, University of Belgrade, Bulevar oslobodjenja 18, 11000 Belgrade, Serbia; tijana.ledina@vet.bg.ac.rs (T.L.); glisic.mica@gmail.com (M.G.); cobanovic.nikola@vet.bg.ac.rs (N.Č.); marija.kovandzic@vet.bg.ac.rs (M.K.); snezab@vet.bg.ac.rs (S.B.)

**Keywords:** lactic acid bacteria (LAB), technological properties, functional properties, probiotics, Serbian cheeses

## Abstract

Owing to the rich diversity of lactic acid bacteria (LAB) microbiota, traditional Serbian white-brined cheeses can serve as a valuable source of LAB strains with promising technological and functional properties. This study aimed to identify potential candidates for developing commercial bacterial cultures, which could be used to produce cheese with distinct sensory qualities and added value as a functional food product. A total of 83 LAB isolates were tested for their ability to grow under different salt concentrations and temperatures; their acidifying, proteolytic, and lipolytic activities; and their production of diacetyl and exopolysaccharides (EPSs). Four strains, one *Lacticaseibacillus paracasei* and three *Lactiplantibacillus plantarum*, were the most promising candidates for further evaluation as adjunct cultures since they showed good resistance to environmental stresses, proteolytic activity, and the ability to produce diacetyl and EPSs. None of the strains was a promising candidate for application as a starter culture or for probiotic use. Further research is required to assess the potential of the isolates to demonstrate desirable characteristics when incorporated into a cheese matrix, primarily focusing on understanding their interaction with the cheese environment and behavior under various processing conditions.

## 1. Introduction

The Balkan Peninsula has a rich tradition of breeding cattle, sheep, and goats, thanks to its favorable geographical location, climate, and abundant meadows and pastures. As a result, milk production has always been essential to the agriculture of the Balkan Peninsula, and dairy products have shaped the region’s culinary heritage [1]. White-brined cheeses are among the most commonly produced and widely consumed cheeses in Serbia [2]. Traditional white-brined cheeses in Serbia are manufactured in mountain village households, using raw milk and traditional cheese-making techniques without the addition of commercial starter cultures [1]. The cheese-making process starts immediately after milking when the rennet is added to raw, unrefrigerated milk. After approximately one hour, the newly formed curd is cut into cubes and transferred to a cotton (gauze) strainer. The strainer is then tied into a knot and hung on wooden hooks to remove the whey. Whey drainage can also be accomplished by pressing the curd in a strainer, using a wooden board weighed down with a heavy stone on top. Afterward, the curd is cut, dry-salted, and laid in layers into a wooden vat. Brine, made with whey and salted water, is added to the vats to overlay the curd. The cheese-ripening process in brine usually takes one to two months at approx. 14–16 °C [3,4,5].

The distinctive sensory quality of traditional cheeses is attributed to their microbiota, which is shaped by a complex interplay between environmental factors and the technological processes employed during cheese manufacture [6]. Lactic acid bacteria (LAB) constitute part of the microbiota that have the most profound impact on the properties of local cheeses [7,8], either due to their acidifying activity (starter cultures) or by facilitating proteolytic and lipolytic events during cheese ripening as non-starter lactic acid bacteria (NSLAB) [9]. Previous studies on traditional white-brined cheeses from Serbia have revealed that the diversity of LAB species and the composition of the microbiota largely depend on the type of cheese and the stage of ripening. Bacteria from the *Lactobacillaceae* family are dominant microbiota in ripened cheeses, particularly *Lactiplantibacillus plantarum* (*L. plantarum*), *Lacticaseibacillus paracasei* (*L. paracasei*), *Lactiplantibacillus paraplantarum* (*L. paraplantarum*), and *Levilactobacillus brevis* (*L. brevis*), as well as leuconostoc and enterococci [1]. Serbian raw milk cheeses represent a valuable reservoir of LAB strains with significant technological potential, considering the capacity of isolated strains to effectively acidify and coagulate milk, carry out proteolysis, and produce aromatic compounds [10,11,12,13,14].

The global cheese market is projected to grow, with the FAO (Food and Agriculture Organization) predicting an increase in weekly cheese consumption from approximately 25,633 kilotons (kt) in 2023 to around 28,638 kt by 2032. This trend reflects rising consumer demand worldwide for dairy-based foods, especially cheeses [15]. It is also estimated that the global white cheese market will rapidly grow in 2017–2025, mainly based on the consumers’ perception that white cheeses are healthier than other cheese types [16]. The growing demand for this type of cheese drives the need for its production on an industrial scale, but it is also crucial to achieve desirable sensory properties in these products. Microorganisms from natural sources, such as traditional cheese types, have always been a main source to obtain genetically stable strains for industrially important products [17]. Due to their rich microbial biodiversity and the biotechnological potential of the microbiota, Serbian traditional cheeses may provide a rich source of strains that can be used in the food industry. Dairy products are also considered the most important food source of newly discovered probiotic microorganisms [18], and they represent an ideal vehicle for delivering probiotics to consumers’ gastrointestinal tracts [19,20]. The characterization of probiotics includes the assessment of stress tolerance, the ability to adhere to the intestinal mucosa, the ability to inhibit pathogenic microorganisms via different mechanisms, safety assessment, and clinical trials [21]. Previous studies proved that strains isolated from traditional Serbian cheeses demonstrate various probiotic properties in vitro, such as aggregation and pathogen exclusion [22] and immune modulation [23,24,25].

This study aimed to screen the technological and functional characteristics of LAB isolated from traditional Serbian cheeses. The purpose was to identify promising candidates for developing commercial bacterial cultures that could be used to produce cheese with distinct sensory qualities and added value as a functional food product. To the best of our knowledge, no commercial starter culture currently exists for producing white-brined cheese that closely resembles traditional varieties, which are only produced on a small scale.

## 2. Materials and Methods

### 2.1. Bacterial Strains and Growth Conditions

In this study, a total of 83 LAB previously isolated from traditional Serbian cheeses—Homolje cheese (n = 9), Zlatar cheese (n = 17), and Sjenica cheese (n = 12) [26,27] were investigated for their technological and functional properties. Briefly, the LAB isolates were initially subjected to the GTG Rep-PCR fingerprinting method using the (GTG)_5_ primer [28]. The isolates that showed different (GTG)_5_ fingerprints, based on the Dice correlation coefficient, and an average linkage (UPGMA or the unweighted pair group method with arithmetic averages) were considered separate strains. The strains were further identified via matrix-assisted laser desorption/ionization time-of-flight (MALDI-TOF) mass spectrometry (MS) (Bruker Daltonics, Bremen, Germany) with score values of ≥ 2.0 (successful identification at the species level). The isolates belonged to the following species: *L. plantarum* (n = 25), *L. paracasei* (n = 24), *L. brevis* (n = 10), *Leuconostoc mesenteroides* (*L. mesenteroides*) (n = 9), *Lactococcus lactis* (*L. lactis*) (n = 3), *Latilactobacillus curvatus* (*L. curvatus*) (n = 3), *Lentilactobacillus buchneri* (*L. buchneri*) (n = 2), *Pediococcus pentosaceus* (*P. pentosaceus*) (n = 2), *Lentilactobacillus kefiri* (*L. kefiri*) (n = 1), *Lentilactobacillus diolivorans* (*L. diolivorans*) (n = 1), *Loigolactobacillus coryniformis* (*L. coryniformis*) (n = 1), *Lactococcus garviae* (*L. garviae*) (n = 1), and *Leuconostoc pseudomesenteroides (L. pseudomesenteroides)* (n = 1). All isolates were maintained at –80 °C in de Man–Rogosa–Sharpe (MRS) broth (Oxoid, Basingstoke, UK) supplemented with 30% (*v*/*v*) glycerol as a cryoprotective agent, except lactococci, which were maintained at –80 °C in M17 broth (Oxoid) supplemented with 30% (*v*/*v*) glycerol. Before the analyses, all isolates were activated via two successive transfers in the appropriate broth at 30 °C for 24 h.

The reference strains used for the screening of LAB antimicrobial activity—*Escherichia coli* (*E. coli*) ATCC 25922, *Staphylococcus aureus* (*S. aureus*) ATCC 25923, *Pseudomonas aeruginosa* (*P. aeruginosa*) ATCC 27853, *Listeria monocytogenes* (*L. monocytogenes*) ATCC 13932, and *Enterococcus faecalis* (*E. faecalis*) ATCC 29212—were stored –80 °C in Tryptone Soya Broth (Oxoid) supplemented with 30% (*v*/*v*) glycerol. Before the analyses, they were routinely cultured in Tryptone Soya Broth (Oxoid) at 37 °C for 24 h.

### 2.2. Growth at Different Temperatures and NaCl Concentrations

The growth at different temperatures and in the presence of NaCl was analyzed as described by Ribeiro et al. [29]. The ability of isolates to grow at different temperatures was determined in MRS or M17 broth after incubation for 48 h at 4, 15, and 45 °C. Meanwhile, the isolates’ growth in different salt concentrations was determined in MRS or M17 broth supplemented with 2, 6, and 10% (*w*/*v*) NaCl at 30 °C for 24 h. Following incubation, the broths’ optical density (OD_630 nm_) was measured using a spectrophotometer (Cecil 2021, Cecil Instruments, Cambridge, UK). Based on the measured optical density, i.e., their ability to grow at different temperatures and in NaCl, the strains were categorized into three groups: OD_630 nm_ < 0.1, OD_630 nm_ = 0.1–0.5, and OD_630 nm_ > 0.5.

### 2.3. Acidifying Activity

Acidification was assessed by inoculating tubes containing 10 mL of UHT milk with 1% (*v*/*v*) MRS or M17 broth cultures and incubating them at 30 °C overnight. The pH values were measured using a pH meter equipped with a combination electrode (Extech, Boston, MA, USA) at time 0 and after 6, 16, and 24 h of incubation at 30 °C.

### 2.4. Proteolytic and Lipolytic Activity

Extracellular proteolytic activity was determined as described by Franciosi et al. [7], with slight modifications. The isolates were streaked with a loop across the surfaces of skim milk agar (SMA) plates, prepared from nutrient agar (Oxoid) supplemented with 10% skim milk powder. Lipolytic activity was determined by plating the isolates on the surface of tributyrin agar (Oxoid) [30]. The plates were incubated at 30 °C for 72 h under anaerobic conditions (AnaeroGen, Thermo Scientific Oxoid, Waltham, MA, USA). Following incubation, the SMA plates were washed with 1% HCl. Transparent halos around colonies on SMA and tributyrin agar were considered a positive reaction for extracellular proteolytic and lipolytic activity, respectively.

### 2.5. Diacetyl and Exopolysaccharide (EPS) Production

Diacetyl production was determined as described by King [31]. Overnight MRS and M17 broth cultures were inoculated (1% *v*/*v*) in 10 mL of UHT milk and incubated at 30 °C for 24 h. One milliliter of cell suspension was transferred to a glass tube in which 0.5 mL of α-naphtol (1% *w*/*v*) and KOH solution (16% *w*/*v*) were then added. After incubation at 37 °C for 10 min, the formation of a red ring at the top of the tube indicated a positive reaction. Exopolysaccharide production was screened by plating strains on the surface of MRS or M17 agar (Oxoid) containing 2% (*w*/*v*) of each of the following carbon sources: glucose, sucrose, and lactose (Himedia, Mumbai, India). Following incubation at 30 °C for 3–5 days under anaerobic conditions, the strains were examined using the loop touch test. The strains that produced mucoid or ropy colonies were considered EPS-positive reactions [32].

### 2.6. Functional Properties

#### 2.6.1. Acidic Resistance and Bile Salt Tolerance

The acidic resistance of the isolates was assessed by inoculating 1% (*v*/*v*) overnight broth cultures into MRS or M17 broth, which had been acidified to pH 2.0 using 1M HCl [33]. Aliquots were withdrawn at time 0 and at 3 h after incubation at 37 °C under acidic conditions, reflecting the time that food usually spends in the stomach. Isolates with a survival rate of higher than 90% were selected for testing bile salt tolerance [34]. The bile salt tolerance of the isolates was tested in MRS or M17 broth supplemented with 0.5% and 1.5% (*w*/*v*) bile salts (HiMedia). The broth was inoculated with 1% (*v*/*v*) overnight cultures and then incubated for 6 h at 37 °C. The total viable counts were enumerated by pour-plating decimal dilutions of aliquots with MRS or M17 agar and after incubation for 48 h at 30 °C under anaerobic conditions. The survival rates were calculated according to the following equation [34]:

$$Survival\;rate=\frac{log\;CFU\;N1}{log\;CFU\;N0}\times 100$$

where N1 represents the total viable count after 3 h at pH 2 or after 6 h in the presence of 0.5% and 1.5% bile salts, and N0 represents the total viable count at time 0.

#### 2.6.2. Safety Assessment: Hemolytic Activity and Gelatinase Production

The hemolytic activity of the isolates was tested on Columbia blood agar with 5% sheep blood (Becton Dickinson GmbH, Heidelberg, Germany). The isolates were plated on the agar surface by streaking with a sterile loop, incubated anaerobically at 30 °C for 48 h, and subsequently investigated for the presence of α-hemolysis or β-hemolysis (green or clear zones around colonies, respectively) [35]. The gelatinase activity was investigated on gelatine agar plates, which consisted of 30 g/L of gelatine (HiMedia), 5 g/L of peptone (HiMedia), 3 g/L of yeast extract (HiMedia), and 17 g/L of agar (HiMedia) [36]. Following incubation (30 °C/48 h), the plates were flooded with saturated ammonium sulfate. The presence of a clear zone around the colonies indicated a positive reaction.

#### 2.6.3. Antagonistic Activity

The antagonistic effect of LAB was evaluated according to Tagg and McGiven [37] using an agar well diffusion assay with modifications. Namely, overnight LAB broth cultures (grown in MRS or M17 broth at 30 °C) were adjusted to pH 6.5 with 1M NaOH to rule out the inhibitory effect of organic acids on the reference strains. The neutralized broth cultures were then centrifuged at 4500 g for 20 min (FastGene HighSpeed Ng003, Nippon Genetics Europe, Düren, Germany), and the supernatants were sterilized with 0.22 µm membrane filters (Filter Lab, Barcelona, Spain) to obtain cell-free supernatants (CFSs). The antimicrobial spectrum of each CFS was tested against *E. coli* ATCC 25922, *S. aureus* ATCC 25923, *P. aeruginosa* ATCC 27853, *L. monocytogenes* ATCC 13932, and *E. faecalis* ATCC 29212. Plate count agar (200 mL) (Oxoid) was inoculated with 100 μL of a suspension of the indicator strains (containing approx. 10^7^ CFU/mL) and poured into Petri dishes. After solidification, wells (5 mm diameter) were punctured in the agar using sterile pipette tips. The wells were filled with 100 µL and left at 4 °C for 4 h to let the CFS diffuse into the agar. The criterion for antimicrobial activity was inhibition zones around the wells of > 1 mm after incubation at 37 °C for 24 h.

### 2.7. Statistical Analysis

The measurements of technological and functional characteristics were performed in triplicate, while safety evaluation tests were performed in duplicate. The relationship between the LAB isolates was determined using hierarchical cluster analysis (HCA) and principal component analysis (PCA) using the XLSTAT™ software 2024.3 (Lumivero 2024, XLSTAT statistical and data analysis solution; https://www.xlstat.com/en). The results of the qualitative assays (proteolytic activity, lipolytic activity, and diacetyl and EPS production) were converted into two coded values (0 and 1). The quantitative analysis data (tolerance to NaCl and temperature) were coded with three values (0, 1, and 2) (Table 1). The absolute values for the acidification capacity and bile salt tolerance were used as the input data. The LAB isolates, numbered from 1 to 83, were classified by species, with nine strains remaining unclassified (Table 2). PCA was performed using varimax rotation, considering the grouped isolates. The Δ pH values were examined for normality and homogeneity of variance by examining the residuals using the coefficients of skewness, kurtosis, and the Shapiro–Wilk normality test. The Kruskal–Wallis test was used for intergroup comparisons, where statistical significance was set at *p* ≤ 0.05.

## 3. Results and Discussion

### 3.1. Growth at Different Temperatures and NaCl Concentrations

In this study, LAB isolates from traditional Serbian cheeses were tested against salt concentrations of 4, 6, and 10% NaCl (Table 2).

All the LAB isolates showed the ability to grow in 4% salt. A salt concentration of 6% had a selective effect on some isolates, depending on the species. All isolates belonging to *L. paracasei* and *L. plantarum* demonstrated good growth at this concentration. In contrast, 6% NaCl inhibited many strains of *L. mesenteroides* and lactococci. Specifically, six *Leuconostoc* isolates (60%) and all lactococci, except for one *L. garviae*, had an OD_630 nm_ value in the range 0.1–0.5 in the presence of 6% NaCl. Meanwhile, the addition of 10% NaCl negatively affected the growth of most isolates; they continued to grow, although at a slower rate. However, one *L. garviae*, one *L. lactic*, and three *L. paracasei* strains were completely inhibited by this highest salt concentration. These results are consistent with those of Ferrari et al. [34], who found that LAB isolated from goat dairies could grow in 4 and 6.5% salt. However, our findings contrast with those of Nicosia et al. [38], which reported *L. plantarum’s* weak ability to grow in 10% salt, since the only four isolates in our study that were unaffected by this salt concentration belonged to the *L. plantarum* species.

Autochthonous LAB face harsh environmental conditions and different stresses during cheese ripening. As a result, they must develop stress responses, which can vary in effectiveness depending on the species and strains of LAB [39]. When selecting wild-type LAB for industrial use, it is crucial to target strains that possess desirable technological properties and can grow in challenging conditions, such as high salt concentrations, low moisture, and varying temperatures.

Osmotolerance is one of the main selection criteria for the technological use of bacterial strains [40]. LAB must tolerate a wide range of salt concentrations since they are often subjected to high salt concentrations during brining and ripening [38]. The salt concentration in traditional Serbian white-brined cheeses may be as high as 10% (*w*/*w*) [10].

### 3.2. Acidifying Activity

In this study, we assessed the ability of isolates to decrease the pH of UHT skim milk after 6, 16, and 24 h (Table 2; Supplementary Table S1). Lactococci acidified milk at the fastest rate, with the pH dropping to an average of 6.10 ± 0.11 after 6 h, a decrease of approximately 0.497. This was followed by *Leuconostoc* isolates, which lowered the pH to 6.15 ± 0.14 after 6 h, an average drop of 0.461. Bacteria from the *Lactobacillaceae* family exhibited slower acidification rates, with average pH reductions of 0.165 for *L. brevis*, 0.217 for *L. paracasei*, 0.268 for *L. plantarum*, and 0.240 for *L. curvatus* after 6 h of incubation. Suitable LAB starters intended for cheese production should be able to decrease the pH value of the milk below 5.3 after 6 h at 30 °C [41]. None of the isolates included in this could fulfill this requirement. These results are consistent with findings from other studies, where most of the LAB isolated from autochthonous cheeses did not meet this criterion [42,43,44]. These results were expected for the members of *Lactobacillaceae* family, as they are known to metabolize lactose slowly [45]. Lactobacilli mainly constitute NSLAB (non-starter lactic acid bacteria) microbiota, which typically exhibits moderate–slow acidifying activity [46]. In strains used as adjunct cultures, good acidification may be considered undesirable since over-acidification can negatively affect cheese yield, rheology, and sensory acceptability [39].

The largest drop in the pH value after 16 and 24 h was reported in *L. mesenteroides*, followed by the lactococci. The average pH values after 16 and 24 h were 4.30 ± 0.61 and 3.75 ± 0.66 for *L. mesenteroides* and 4.86 ± 0.10 and 4.19 ± 0.84 for lactococci, respectively. After 16 h of incubation, *L. mesenteroides* strains exhibited a significantly greater decrease in pH compared with the other groups (*p* < 0.05). After 24 h, this decrease was significantly greater compared with *L. brevis* and *L. plantarum* (Supplementary Table S1). The pH value after 24 h was higher than that after 16 h in five *L. brevis* strains, two *L. mesenteroides* strains, one *L. buchneri* strain, one *L. curvatus* strain, and one *P. pentosaceus* strain.

The acidification rate, according to our results, could also be considered a species-specific feature rather than a strain-specific feature. Our results do not agree with the results of previous studies, where faster- or slower-acidifying strains were detected within the same species, proving that, in these cases, the acidifying capacity was strain-specific [29,45,47].

### 3.3. Proteolytic and Lipolytic Activity

In this study, *L. plantarum* isolates most commonly demonstrated proteolytic activity (n = 9 (36%)), followed by *L. brevis* (n = 3 (30%)) and *L. paracasei* (n = 6 (24%)) isolates (Table 2). The proteolytic activity of members of the *Lactobacillaceae* family can be explained by the fact that they have a limited amino acid biosynthetic capacity, which they compensate for with numerous peptidases, amino acid permeases, and multiple oligopeptide transport systems [48]. Proteinase PrtP was previously identified in *L. paracasei* and *L. plantarum* isolates obtained from Serbian cheeses [14,49,50].

Lipolytic activity plays a key role in the aroma development of cheese because the metabolites produced during lipolysis, even in small amounts, contribute to the flavor and serve as substrates for other catabolic processes [51]. Since excessive lipolysis can result in sensory defects [39], an appropriate candidate for the selection of adjunct cultures should not exhibit undesirable lipolytic activity [45,52]. Our research findings indicate that lipolytic activity is not a common trait among LAB found in traditional Serbian cheeses, as only two isolates—one *L. brevis* and one *L. lactis*—exhibited lipolytic activity (Table 2). These results are consistent with those of other studies, which also suggest that lipolytic activity is not a common characteristic among LAB isolated from cheese varieties [29,38,45,52,53,54].

### 3.4. EPS and Diacetyl Production

In our research, colonies that produced ropy colonies on MRS agar supplemented with different sugars as a carbon source were considered positive for EPS production. *L. plantarum* isolates were identified as the most proficient EPS producers, with 12 isolates (48.0%) testing positive for EPS production. *L. paracasei* followed with five isolates (20.8%). Additionally, one isolate each of *L. brevis, L. curvatus,* and *P. pentosaceus* was found to produce EPSs. These results align with Margalho et al.’s findings [55], reporting *L. plantarum* as the primary EPS producer among LAB from Brazilian artisanal cheeses. However, our findings differ from most studies on LAB from cheese, where EPS production was typically observed as a rare trait [29,52,53,54]. Exopolysaccharide production is a valuable technological characteristic in LAB used for cheesemaking since EPSs can enhance the texture (by increasing viscosity), rheological properties, and sensory properties of cheese [52].

In cheesemaking, the drop in pH during the acidification process enables LAB cells to absorb citrate from milk, which is then used to produce aromatic compounds such as diacetyl [56]. Even at low concentrations, diacetyl gives a buttery aroma to dairy products, making them more appealing to consumers [34]. Therefore, LAB strains that can produce diacetyl are considered advantageous as adjunct cultures for some cheese types [57]. In addition, diacetyl has inhibitory activity against some food-borne pathogens and can act as a biopreservative [58]. Most of the strains analyzed in this study (n = 54 (64.3%)) demonstrated the capacity to synthesize diacetyl, particularly those belonging to the *Lactobacillaceae* family. Among those strains, 80.0% of *L. brevis* isolates (n = 8), 76.0% of *L. plantarum* isolates (n = 19), and 70.8% of *L. paracasei* isolates (n = 17) could produce diacetyl (Table 2). Diacetyl production is a seemingly common trait in *L. paracasei* from dairy environments [54]. *Leuconostoc* spp. in pure cultures does not produce diacetyl since pyruvate formed during citrate metabolism is transformed into lactate rather than diacetyl and acetoin. However, *Leuconostoc* spp. can produce diacetyl and acetoin when the pH level falls below 5.5, possibly because of a reduced ability to absorb lactose [59]. Our results show that only three *L. mesenteroides* isolates produced diacetyl, which is consistent with findings from other studies that also characterized them as uncommon diacetyl producers [53,54].

### 3.5. Functional Properties

#### 3.5.1. Safety Evaluation: Hemolytic Activity and Gelatinase Production

Hemolytic and gelatinase activities are important factors in evaluating the safety of LAB strains, as strains exhibiting these traits may present health risks [54,55]. In our study, none of the strains exhibited hemolytic activity (Table 2). Hemolytic activity appears to be infrequent among LAB, as most studies conducted on LAB strains from dairy environments have reported its absence [29,34,35,49].

None of the isolates exhibited gelatinase activity (Table 2), which is consistent with the results of other studies where gelatinase activity in LAB was found exclusively in enterococci [29,60].

#### 3.5.2. Acidic Resistance and Bile Salt Tolerance

**Figure 1:**
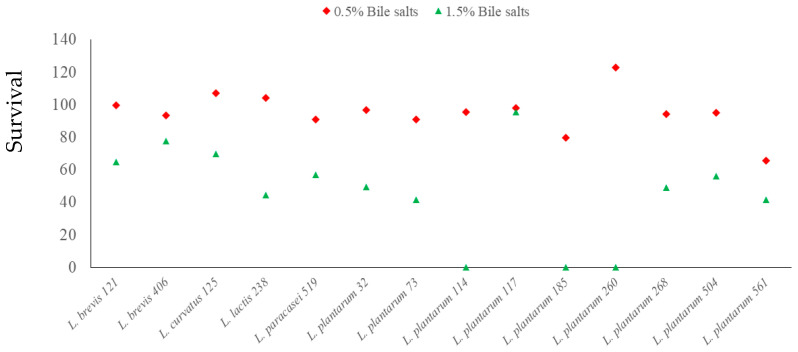
Survival of LAB isolates at pH 2.5 and with 0.5% and 1.5% bile salts added after 6 h of incubation. Points are the survival rate, where N1 is the number of bacteria after 6 h, and N0 is the number of bacteria in the initial inoculum.

According to Ferrari et al.’s research [34], a survival rate exceeding 90% was applied as a pre-selection criterion for the probiotic characterization of LAB. In this study, 14 out of 83 isolates (16.87%) demonstrated a survival rate of over 90% at pH 2.0, indicating good acid resistance [61]. The acid-resistant isolates belonged to the following species: *L. plantarum* (n = 9), *L. brevis* (n = 2), *L. paracasei* (n = 1), *L. curvatus* (n = 1), and *L. lactis* (n = 1). Consequently, they were selected to further test their ability to survive in the presence of bile salts (Figure 1). In the presence of 0.5% bile salts, none of the isolates were completely inhibited, and three of the isolates (one *L. lactis*, *L. plantarum*, and *L. curvatus* each) could grow to higher counts. A concentration of 1.5% bile salts inhibited growth in most of the isolates, with three *L. plantarum* isolates being completely inhibited. These results differ from those of Ferrari et al. [34] and de Almeida Júnior et al. [61], where the survival rate of LAB in the presence of bile salts exceeded 95% for all isolates. While a higher bile salt concentration (2% *w*/*v*) was used in those studies, the incubation period was only 3 h, which is half the duration used for bile salt exposure in this study. Monteagudo-Mera et al. [62] also observed high viability of lactobacilli in the presence of bile salts, although the highest bile salt concentration tested was 0.4% over a 4 h incubation period.

Although this study evaluates acid and bile-salt tolerance as critical probiotic characteristics, other important characteristics, such as adhesion to intestinal epithelial cells, immunomodulation, or pathogen inhibition in cell lines or animal models, were not tested, and they would require further, more in-depth, analyses.

The viability of isolates to survive in the presence of 0.5% and 1.5% bile salts is shown in Figure 1.

#### 3.5.3. Antimicrobial Activity

Since none of the neutralized CFSs showed antimicrobial activity, the antimicrobial compounds were not assessed further. Antimicrobial activity, especially due to proteinaceous substances, is a rare outcome in LAB isolated from cheese [8,52]. Acid production is the primary inhibitory mechanism of LAB isolates, as supported by other studies showing that most isolates lose their inhibitory effect once the pH is adjusted to neutral [43].

### 3.6. Principal Component Analysis

**Figure 2:**
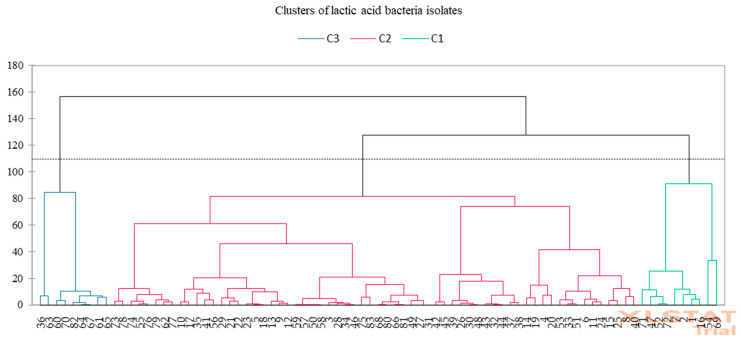
Cluster analysis run of LAB isolates from cheese. The results of technological characterization assays were used as input variables.

Based on technological and functional characteristics using hierarchical cluster analysis, the 83 LAB isolates were divided into three homogeneous groups via a dissimilarity approach. The first, second, and third clusters included 64, 10, and 9 isolates, respectively (Figure 2).

**Figure 3:**
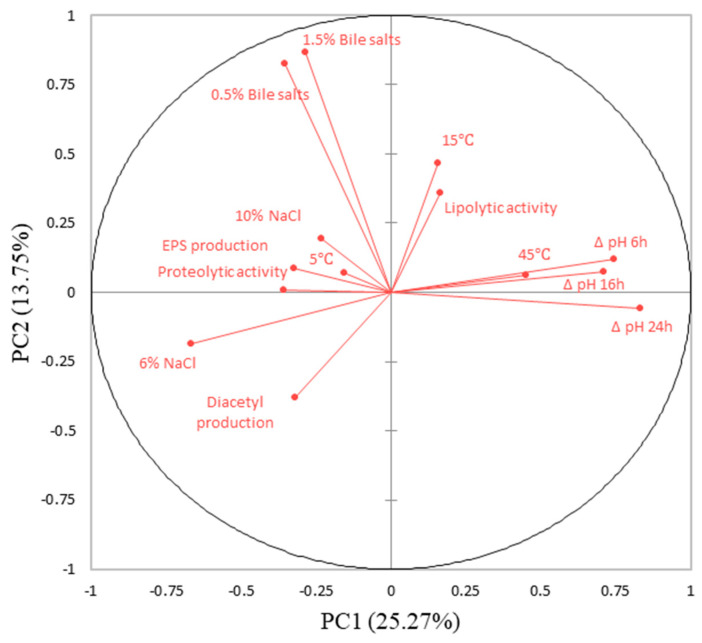
Principal component analysis (PCA) of technological and probiotic characteristics of LAB isolates: projection of the variables.

**Figure 4:**
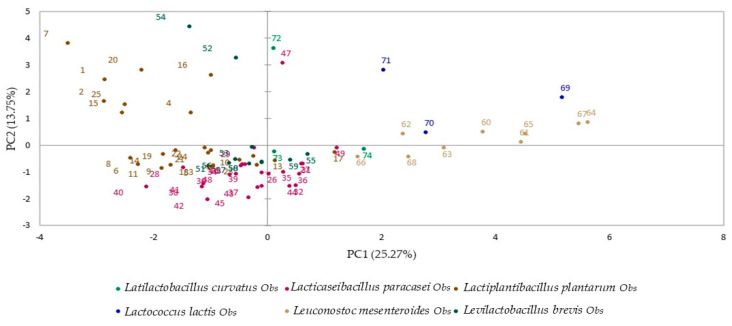
Principal component analysis (PCA) of technological characterization assays of LAB isolates: projection of the 74 isolates in the space of PC1 and PC2.

The cluster analysis indicated the isolates with similar technological traits and enzymatic activity. However, since cluster analysis does not highlight the variables responsible for their similarities or dissimilarities [63], a multivariate approach comprising PCA was used. The PCA revealed a percentage of variability of 39.02%, where PC1 and PC2 accounted for 25.27% and 13.75%, respectively (Figure 3 and Figure 4). The correlation analysis between the isolates’ technological and functional characteristics suggested a nearly homogeneous distribution of variables on the plane of principal components (Figure 3). The acidification potential after 6, 16, and 24 h was correlated with the ability to grow at 45 and 15 °C and was inversely correlated with diacetyl production, EPS production, growth in 6% NaCl, and proteolytic activity. Tolerance to bile salts and hemolytic and lipolytic activity were positively correlated with diacetyl production and growth in 6% NaCl, respectively. Furthermore, three main groups of isolates were identified based on the position of the variables in the factorial space of the PCA (Figure 4). Isolates grouped in cluster 3, mostly including *L. mesenteroides* species, located in the lower and upper right quadrants, had better results regarding their acidification potential and growth at 45 °C. Isolates grouped in cluster 2 and located in both upper quadrants, especially the subgroup of isolates coded 52, 54, 72, 47, and 16 (*L. brevis* 121, *L. brevis* 406, *L. curvatus* 125, *L. paracasei* 519, and *L. plantarum* 268), showed better results in the bile salt tolerance experiments. Finally, the isolates grouped in the largest cluster 1 were mostly located in the lower left quadrant, slightly in the negative axis of PC2, and had better results regarding diacetyl production and salt concentration tolerance. In addition, the *L. lactis* 27 isolate showed lipolytic activity but without bile salt tolerance. Aligning with previous studies [56,64,65], the technological and functional properties of the tested LAB from cheeses were strain-specific, without an observed pattern that could be related to all isolates within the same species.

The main limitation of this study lies in its exclusive reliance on in vitro testing, using a series of indicator measurements rather than in situ testing within a cheese matrix under conditions that simulate industrial production.

Additionally, in this study, we focused on simple and rapid screening methods, which provide a cost-effective way to assess the main characteristics of a larger number of strains, while the application of in-depth analyses, such as strain-specific quantification of acid production and analyses of metabolic and enzymatic activity, could contribute to the optimization of strain selection.

While this study does not replicate key aspects of industrial cheese production, such as the use of pasteurized milk, it provides valuable insights into the population of “wild-type” lactic acid bacteria isolated from natural habitats. These findings serve as an important foundation for the preliminary characterization of commercially applicable microorganisms, especially adjunct cultures. Traditional cheese types, as a source of LAB populations with rich diversity, offer an excellent starting point for the development of industrial cultures. Testing the technological properties of LAB in vitro represents a crucial first step in this process.

## 4. Conclusions

This research represents a preliminary study aimed at isolating LAB strains with the potential for commercial applications using cost-effective and robust methods. According to this study’s findings, the LAB strains isolated from traditional Serbian cheeses show great potential for use in cheese production. Although they are not ideal as starter cultures due to their limited acidification ability, they exhibit other valuable characteristics, including tolerance to high salt concentrations, growth at typical cheese-ripening temperatures, protein degradation, and diacetyl and EPS production. Consequently, these strains hold promise as adjunct cultures. Specifically, four strains—*L. paracasei* 434 and *L. plantarum* 157, 260, and 561—are the most promising candidates for further evaluation as adjunct cultures. For potential future commercial applications, further research is required to assess whether these isolates demonstrate desirable characteristics when incorporated into a cheese matrix. This includes evaluating their capacity to survive, grow, and interact effectively with the complex conditions present during cheese ripening. Understanding their adaptability and functional contributions to the ripening environment will be essential in determining their potential for commercial use in cheese production. Understanding how these isolates interact with the cheese environment, such as their behavior under different processing conditions, will be critical to determining their suitability for improving the quality of the cheese. Isolates’ proteolytic activity, along with EPS and diacetyl production, can play a key role in achieving industrially produced cheese with sensory characteristics comparable to traditional cheese.

## Figures and Tables

**Table 1:** Qualitative codes used for multivariate analysis (hierarchical cluster analysis and principal component analysis).

Qualitative Code	Growth at 45, 15, and 5 °C	Growth with 6 and 10% NaCl	Proteolytic Activity	Lipolytic Activity	Diacetyl Production	EPS Production
0	<0.1	<0.1	Negative	Negative	Negative	Negative
1	0.1–0.5	0.1–0.5	Positive	Positive	Positive	Positive
2	>0.5	>0.5	-	-	-	-

-: Not applicable.

**Table 2:** Technological properties of the LAB isolates.

No.	Isolate	Δ pH			Temperature			NaCl		D.P.	P.A.	L.A.	EPS P.
		6 h	16 h	24 h	45 °C	15 °C	5 °C	6%	10%				
	*L. plantarum* (n = 25)												
1	*L. plantarum* 32	0.10	0.76	0.99	0.1–0.5	>0.5	0.1–0.5	>0.5	0.1–0.5	-	+	-	-
2	*L. plantarum* 73	0.10	0.53	0.85	>0.5	>0.5	0.1–0.5	>0.5	0.1–0.5	+	+	-	-
3	*L. plantarum* 111	0.23	0.85	1.09	>0.5	>0.5	0.1–0.5	>0.5	0.1–0.5	+	-	-	-
4	*L. plantarum* 114	0.39	0.90	0.92	>0.5	>0.5	0.1–0.5	>0.5	>0.5	+	-	-	-
5	*L. plantarum* 144	0.33	0.88	1.17	0.1–0.5	>0.5	0.1–0.5	>0.5	0.1–0.5	+	-	-	-
6	*L. plantarum* 157	0.20	0.86	1.09	0.1–0.5	>0.5	0.1–0.5	>0.5	0.1–0.5	+	+	-	+
7	*L. plantarum* 177	0.09	0.34	0.77	0.1–0.5	>0.5	0.1–0.5	>0.5	0.1–0.5	-	-	-	+
8	*L. plantarum* 185	0.19	0.93	1.13	0.1–0.5	0.1–0.5	0.1–0.5	>0.5	0.1–0.5	+	+	-	-
9	*L. plantarum* 207	0.17	0.95	1.30	0.1–0.5	>0.5	0.1–0.5	>0.5	0.1–0.5	+	-	-	+
10	*L. plantarum* 208	0.51	1.00	1.12	>0.5	>0.5	0.1–0.5	>0.5	0.1–0.5	+	-	-	+
11	*L. plantarum* 210	0.22	0.77	1.01	0.1–0.5	>0.5	0.1–0.5	>0.5	0.1–0.5	+	+	-	-
12	*L. plantarum* 217	0.38	1.34	1.73	0.1–0.5	>0.5	0.1–0.5	>0.5	0.1–0.5	+	-	-	+
13	*L. plantarum* 219	0.24	2.03	2.6	0.1–0.5	>0.5	0.1–0.5	>0.5	0.1–0.5	+	-	-	+
14	*L. plantarum* 223	0.33	0.88	0.88	0.1–0.5	>0.5	0.1–0.5	>0.5	>0.5	+	+	-	-
15	*L. plantarum* 260	0.26	0.87	1.03	0.1–0.5	>0.5	0.1–0.5	>0.5	0.1–0.5	+	+	-	+
16	*L. plantarum* 268	0.30	1.29	1.31	0.1–0.5	>0.5	0.1–0.5	>0.5	0.1–0.5	-	-	-	-
17	*L. plantarum* 273	0.54	1.87	2.12	>0.5	>0.5	0.1–0.5	>0.5	0.1–0.5	+	-	-	+
18	*L. plantarum* 283	0.26	1.00	1.11	0.1–0.5	>0.5	0.1–0.5	>0.5	0.1–0.5	+	-	-	-
19	*L. plantarum* 503	0.34	0.93	0.94	0.1–0.5	>0.5	0.1–0.5	>0.5	>0.5	+	-	-	+
20	*L. plantarum* 504	0.23	0.90	1.09	>0.5	>0.5	0.1–0.5	>0.5	>0.5	+	-	-	+
21	*L. plantarum* 527	0.19	0.77	0.95	0.1–0.5	>0.5	0.1–0.5	>0.5	0.1–0.5	-	-	-	-
22	*L. plantarum* 536	0.27	0.84	0.97	0.1–0.5	>0.5	0.1–0.5	>0.5	0.1–0.5	-	-	-	+
23	*L. plantarum* 548	0.29	1.25	1.84	0.1–0.5	>0.5	0.1–0.5	>0.5	0.1–0.5	+	-	-	-
24	*L. plantarum* 554	0.26	0.97	1.06	0.1–0.5	>0.5	0.1–0.5	>0.5	0.1–0.5	-	+	-	-
25	*L. plantarum* 561	0.28	0.83	0.78	0.1–0.5	>0.5	0.1–0.5	>0.5	0.1–0.5	+	+	-	+
	*L. paracasei* (n = 24)												
26	*L. paracasei* 116	0.22	1.26	2.22	0.1–0.5	0.1–0.5	0.1–0.5	>0.5	0.1–0.5	-	-	-	-
27	*L. paracasei* 155	0.26	1.30	2.34	>0.5	>0.5	0.1–0.5	>0.5	0.1–0.5	+	-	-	-
28	*L. paracasei* 180	0.14	0.36	0.54	>0.5	>0.5	0.1–0.5	>0.5	0.1–0.5	+	-	-	-
29	*L. paracasei* 195	0.24	0.55	1.96	>0.5	>0.5	0.1–0.5	>0.5	0.1–0.5	-	-	-	+
30	*L. paracasei* 224	0.20	0.86	1.54	0.1–0.5	0.1–0.5	0.1–0.5	>0.5	0.1–0.5	-	-	-	-
31	*L. paracasei* 293	0.24	1.29	2.39	>0.5	>0.5	0.1–0.5	>0.5	0.1–0.5	+	-	-	-
32	*L. paracasei* 304	0.25	1.33	2.39	>0.5	0.1–0.5	0.1–0.5	>0.5	0.1–0.5	+	-	-	-
33	*L. paracasei* 306	0.25	0.83	1.84	>0.5	>0.5	0.1–0.5	>0.5	0.1–0.5	+	+	-	-
34	*L. paracasei* 307	0.23	0.59	1.37	>0.5	>0.5	0.1–0.5	>0.5	0.1–0.5	+	-	-	-
35	*L. paracasei* 403	0.22	1.02	2.15	>0.5	>0.5	0.1–0.5	>0.5	<0.1	+	-	-	+
36	*L. paracasei* 404	0.23	1.22	2.26	0.1–0.5	>0.5	<0.1	>0.5	0.1–0.5	+	-	-	-
37	*L. paracasei* 409	0.24	1.05	2.35	>0.5	0.1–0.5	0.1–0.5	>0.5	0.1–0.5	+	+	-	-
38	*L. paracasei* 412	0.22	0.7	1.05	>0.5	0.1–0.5	0.1–0.5	>0.5	0.1–0.5	+	+	-	-
39	*L. paracasei* 413	0.19	0.74	1.62	>0.5	0.1–0.5	0.1–0.5	>0.5	0.1–0.5	-	-	-	-
40	*L. paracasei* 434	0.16	0.63	1.21	0.1–0.5	0.1–0.5	0.1–0.5	>0.5	0.1–0.5	+	+	-	+
41	*L. paracasei* 441	0.19	0.69	1.10	>0.5	0.1–0.5	0.1–0.5	>0.5	0.1–0.5	+	-	-	+
42	*L. paracasei* 444	0.22	0.73	1.28	0.1–0.5	0.1–0.5	0.1–0.5	>0.5	<0.1	+	+	-	-
43	*L. paracasei* 446	0.19	0.84	1.85	>0.5	0.1–0.5	0.1–0.5	>0.5	0.1–0.5	+	-	-	-
44	*L. paracasei* 448	0.23	1.09	2.26	>0.5	0.1–0.5	0.1–0.5	>0.5	0.1–0.5	+	-	-	-
45	*L. paracasei* 449	0.22	0.73	1.60	>0.5	0.1–0.5	0.1–0.5	>0.5	<0.1	+	+	-	-
46	*L. paracasei* 452	0.20	0.67	1.10	>0.5	>0.5	0.1–0.5	>0.5	0.1–0.5	+	-	-	-
47	*L. paracasei* 519	0.28	1.47	2.34	>0.5	>0.5	0.1–0.5	>0.5	0.1–0.5	-	-	-	+
48	*L. paracasei* 521	0.19	0.81	1.31	0.1–0.5	0.1–0.5	0.1–0.5	>0.5	0.1–0.5	-	-	-	-
49	*L. paracasei* 522	0.21	1.37	2.34	>0.5	>0.5	0.1–0.5	>0.5	0.1–0.5	-	-	-	-
	*L. brevis* (n = 10)												
50	*L. brevis* 112	0.15	1.35	0.94	>0.5	>0.5	0.1–0.5	>0.5	0.1–0.5	+	-	-	-
51	*L. brevis* 113	0.18	0.94	1.03	>0.5	>0.5	0.1–0.5	>0.5	0.1–0.5	+	+	-	-
52	*L. brevis* 121	0.15	1.41	1.02	>0.5	>0.5	0.1–0.5	>0.5	0.1–0.5	-	-	-	-
53	*L. brevis* 228	0.14	1.21	0.93	>0.5	>0.5	0.1–0.5	>0.5	0.1–0.5	-	+	-	-
54	*L. brevis* 406	0.16	0.72	0.92	>0.5	>0.5	0.1–0.5	>0.5	0.1–0.5	+	+	+	-
55	*L. brevis* 411	0.16	0.86	1.11	>0.5	>0.5	0.1–0.5	0.1–0.5	0.1–0.5	+	-	-	-
56	*L. brevis* 455	0.19	0.89	1.01	>0.5	>0.5	0.1–0.5	>0.5	0.1–0.5	+	-	-	+
57	*L. brevis* 510	0.15	0.85	1.00	>0.5	>0.5	0.1–0.5	>0.5	0.1–0.5	+	-	-	-
58	*L. brevis* 556	0.13	1.23	0.97	>0.5	>0.5	0.1–0.5	>0.5	0.1–0.5	+	-	-	-
59	*L. brevis* 558	0.24	1.34	1.10	>0.5	>0.5	0.1–0.5	>0.5	0.1–0.5	+	-	-	-
	*L. mesenteroides* (n = 9)												
60	*L. mesenteroides* 54	0.44	1.83	3.01	>0.5	>0.5	0.1–0.5	0.1–0.5	0.1–0.5	-	-	-	-
61	*L. mesenteroides* 59	0.44	2.93	3.32	>0.5	>0.5	0.1–0.5	0.1–0.5	0.1–0.5	+	-	-	-
62	*L. mesenteroides* 102	0.25	1.69	1.66	>0.5	>0.5	0.1–0.5	0.1–0.5	0.1–0.5	-	-	-	-
63	*L. mesenteroides* 105	0.42	2.00	2.42	>0.5	>0.5	<0.1	>0.5	0.1–0.5	-	-	-	-
64	*L. mesenteroides* 171	0.70	2.91	3.35	>0.5	>0.5	0.1–0.5	0.1–0.5	0.1–0.5	-	-	-	-
65	*L. mesenteroides* 179	0.52	3.27	3.54	>0.5	>0.5	0.1–0.5	>0.5	0.1–0.5	-	-	-	-
66	*L. mesenteroides* 241	0.35	1.77	1.83	>0.5	>0.5	0.1–0.5	>0.5	0.1–0.5	+	-	-	-
67	*L. mesenteroides* 255	0.65	2.81	3.36	>0.5	>0.5	0.1–0.5	0.1–0.5	0.1–0.5	-	-	-	-
68	*L. mesenteroides* 285	0.38	1.95	2.93	>0.5	>0.5	0.1–0.5	>0.5	0.1–0.5	+	-	-	-
	*L. lactis* (n = 3)												
69	*L. lactis* 27	0.62	1.86	3.39	>0.5	>0.5	0.1–0.5	0.1–0.5	<0.1	-	+	+	-
70	*L. lactis* 95	0.45	1.68	1.94	0.1–0.5	>0.5	0.1–0.5	0.1–0.5	0.1–0.5	-	-	-	-
71	*L. lactis* 238	0.42	1.69	1.92	>0.5	>0.5	0.1–0.5	0.1–0.5	0.1–0.5	+	-	-	-
	*L. curvatus* (n = 3)												
72	*L. curvatus* 125	0.27	1.37	1.11	>0.5	>0.5	0.1–0.5	>0.5	0.1–0.5	-	-	-	-
73	*L. curvatus* 242	0.08	0.61	0.97	>0.5	>0.5	0.1–0.5	0.1–0.5	0.1–0.5	+	-	-	+
74	*L. curvatus* 243	0.37	0.87	1.17	>0.5	>0.5	0.1–0.5	0.1–0.5	0.1–0.5	+	-	-	-
75	*L. buchneri* 76	0.46	1.27	1.05	>0.5	>0.5	0.1–0.5	>0.5	0.1–0.5	-	-	-	-
76	*L. buchneri* 405	0.03	1.09	1.12	>0.5	>0.5	0.1–0.5	0.1–0.5	0.1–0.5	+	-	-	-
77	*P. pentosaceus* 267	0.06	1.12	0.95	>0.5	>0.5	0.1–0.5	0.1–0.5	0.1–0.5	-	-	-	-
78	*P. pentosaceus* 270	0.17	0.47	0.64	0.1–0.5	>0.5	0.1–0.5	0.1–0.5	0.1–0.5	+	-	-	+
79	*L. kefiri* 147	0.14	0.91	0.85	0.1–0.5	>0.5	0.1–0.5	0.1–0.5	0.1–0.5	-	-	-	-
80	*L. diolivorans* 509	0.38	2.00	2.48	>0.5	>0.5	0.1–0.5	>0.5	0.1–0.5	+	-	-	-
81	*L. coryniformis* 417	0.25	1.64	1.72	>0.5	>0.5	0.1–0.5	>0.5	0.1–0.5	+	-	-	-
82	*L. garviae* 44	0.69	2.18	2.69	>0.5	>0.5	0.1–0.5	0.1–0.5	0.1–0.5	-	-	-	-
83	*L. pseudomesenteroides* 309	0.24	1.04	1.00	>0.5	>0.5	0.1–0.5	>0.5	<0.1	-	-	-	-

D.P.—diacetyl production; P.A.—proteolytic activity; L.A.—lipolytic activity; EPS P.—exopolysaccharide production; *L. plantarum*—*Lactiplantibacillus plantarum*; *L. paracasei*—*Lacticaseibacillus paracasei*; *L. brevis*—*Levilactobacillus brevis*; *L. mesenteroides*—*Leuconostoc mesenteroides*; *L. lactis*—*Lactococcus lactis*; *L. curvatus*—*Latilactobacillus curvatus*; *L. buchneri*—*Lentilactobacillus buchneri*; *L. kefiri*—*Lentilactobacillus kefiri*; *P. pentosaceus*—*Pediococcus pentosaceus*; *L. coryniformis*—*Loigolactobacillus coryniformis*; *L. diolivorans*—*Lentilactobacillus diolivorans*; *L. garviae*—*Lactococcus garviae*; *L. pseudomesenteroides*—*Leuconostoc pseudomesenteroides*.

## Data Availability

The original contributions presented in the study are included in the article/Supplementary Materials, further inquiries can be directed to the corresponding author.

## Supplementary Materials

The following supporting information can be downloaded at: https://www.mdpi.com/article/10.3390/foods14010038/s1, Table S1: Origins of the LAB strains (cheese types); Table S2: Acidifying activity of six LAB groups after 6, 16, and 24 h of incubation (mean ± SD).
