# Supplementary material for: Technological and Functional Characteristics of Lactic Acid Bacteria from Traditional Serbian Cheeses
Source: Foods. 2024 Dec 26;14(1):38. doi: 10.3390/foods14010038 (PMC11719953; doi:10.3390/foods14010038)
Supplement: Supplementary file 1 [file foods-14-00038-s001.zip › foods-3395863-supplementary.pdf]

Table S1. Origins of the LAB strains (cheese types).

	**Homolje Cheese (n = 9)**	**Zlatar Cheese (n = 17)**	**Sjenica Cheese (n = 12)**
L. plantarum (n = 25)	4	14	7
L. paracasei (n = 24)	1	8	15
L. brevis (n = 10)	2	2	6
L. mesenteroides (n = 9)	4	5	0
L. curvatus (n = 3)	0	2	1
L. lactis (n = 3)	2	1	0
L. buchneri (n = 2)	1	0	1
P. pentosaceus (n = 2)	0	2	0
L. coryniformis (n = 1)	0	0	1
L. diolivorans (n = 1)	0	0	1
L. kefiri (n = 1)	0	1	0
L. garviae (n = 1)	1	0	0
L. pseudomesenteroides (n = 1)	0	1	0
**Total**	**15**	**36**	**32**

Table S2. Acidifying activity of six LAB groups after 6, 16, and 24 h of incubation (mean ± SD).

**Isolate**	***Levilactobacillus brevis***	***Latilactobacillus curvatus***	***Leuconostoc mesenteroides***	***Lactococcus lactis***	***Lacticaseibacillus paracasei***	***Lactiplantibacillus plantarum***
ΔpH 6 h	0.165 ± 0.032^{a}	0.240 ± 0.147^{ab}	0.461 ± 0.142^{b}	0.497 ± 0.108^{bc}	0.217 ± 0.032^{ac}	0.268 ± 0.111^{bc}
ΔpH 16 h	1.080 ± 0.253^{a}	0.950 ± 0.386^{ab}	2.351 ± 0.616^{b}	1.743 ± 0.101^{ab}	0.922 ± 0.306^{a}	0.982 ± 0.357^{a}
ΔpH 24 h	1.003 ± 0.066^{a}	1.083 ± 0.103^{abc}	2.824 ± 0.696^{b}	2.417 ± 0.843^{b}	1.767 ± 0.548^{bc}	1.194 ± 0.435^{c}

Kruskal–Wallis test; different lower-case superscript letters within the same row indicate significant differences between groups ^{a,b,c} ($p < 0.05$).
